# Evaluation of Trace Elements in Augmentation of Statin-Induced Cytotoxicity in Uremic Serum-Exposed Human Rhabdomyosarcoma Cells

**DOI:** 10.3390/toxins10020053

**Published:** 2018-01-25

**Authors:** Hitoshi Uchiyama, Masayuki Tsujimoto, Naomi Shimada, Koji Tsutsui, Ayaka Nitta, Takuya Yoshida, Taku Furukubo, Satoshi Izumi, Tomoyuki Yamakawa, Hidehisa Tachiki, Tetsuya Minegaki, Kohshi Nishiguchi

**Affiliations:** 1 Research & Development Division, Towa Pharmaceutical Co., Ltd., Kyoto Research Park KISTIC#202, 134 Chudoji Minami-Machi, Shimogyo-ku, Kyoto 600-8813, Japan; h-uchiyama@towayakuhin.co.jp (H.U.); h-tachiki@towayakuhin.co.jp (H.T.); 2 Department of Clinical Pharmacy, Faculty of Pharmaceutical Science, Kyoto Pharmaceutical University, 5 Misasagi Nakauchi-cho, Yamashina-ku, Kyoto 607-8414, Japan; ky09173@poppy.kyoto-phu.ac.jp (N.S.); ky09225@poppy.kyoto-phu.ac.jp (K.T.); ky09267@poppy.kyoto-phu.ac.jp (A.N.); kd13007@poppy.kyoto-phu.ac.jp (T.Y.); t-minegaki@mb.kyoto-phu.ac.jp (T.M.); kohshi@mb.kyoto-phu.ac.jp (K.N.); 3 Department of Pharmacy Service, Shirasagi Hospital, 7-11-23 Kumata, Higashisumiyoshi-ku, Osaka 546-0002, Japan; furukubo@shirasagi-hp.or.jp (T.F.); izumi@shirasagi-hp.or.jp (S.I.); 4 Department of Medicine, Shirasagi Hospital, 7-11-23 Kumata, Higashisumiyoshi-ku, Osaka 546-0002, Japan; yamakawa@shirasagi-hp.or.jp

**Keywords:** end-stage kidney disease, uremic serum, simvastatin, cytotoxicity, zinc, rhabdomyolysis

## Abstract

Patients with end-stage kidney disease (ESKD) are at higher risk for rhabdomyolysis induced by statin than patients with normal kidney function. Previously, we showed that this increase in the severity of statin-induced rhabdomyolysis was partly due to uremic toxins. However, changes in the quantity of various trace elements in ESKD patients likely contribute as well. The purpose of this study is to determine the effect of trace elements on statin-induced toxicity in rhabdomyosarcoma cells exposed to uremic serum (US cells) for a long time. Cell viability, apoptosis, mRNA expression, and intracellular trace elements were assessed by viability assays, flow cytometry, real-time RT-PCR, and ICP-MS, respectively. US cells exhibited greater simvastatin-induced cytotoxicity than cells long-time exposed with normal serum (NS cells) (non-overlapping 95% confidence intervals). Intracellular levels of Mg, Mn, Cu, and Zn were significantly less in US cells compared to that in NS cells (*p* < 0.05 or 0.01). Pre-treatment with TPEN increased simvastatin-induced cytotoxicity and eliminated the distinction between both cells of simvastatin-induced cytotoxicity. These results suggest that Zn deficiencies may be involved in the increased risk for muscle complaints in ESKD patients. In conclusion, the increased severity of statin-induced rhabdomyolysis in ESKD patients may be partly due to trace elements deficiencies.

## 1. Introduction

3-Hydroxy-3-methylglutaryl coenzyme A reductase inhibitors (statins), which are potent inhibitors of cholesterol biosynthesis, are widely used for the prevention of atherosclerotic cardiovascular disease due to hypercholesterolemia [1,2,3]. However, statins are associated with a variety of muscle complaints such as myopathies and rhabdomyolysis. Although Strippoli et al. reported that the adverse drug reaction profile of statins was similar to that of placebo in patients with chronic kidney disease [4], rhabdomyolysis is a rare adverse drug reaction and statin-induced rhabdomyolysis was partly regarded as problematic in patients with end-stage kidney disease (ESKD). It is thought that one of the risk factors for rhabdomyolysis among statin users may be, while not statistically significant, kidney dysfunction [5]. The frequency of rhabdomyolysis in ESKD patients may be higher than that in patients with normal kidney function. Recently, we reported that uremic toxins accumulated in ESKD patients is, at least in part, associated with enhancement of statin-induced cytotoxicity via small G-protein geranylgeranylation [6]. In statin users, severe vitamin D deficiency that occurs in ESKD patients also appear to be one of risk factors for myalgia which is an initial symptom of rhabdomyolysis [7,8]. These facts indicate that various factors is associated with risk enhancement of statin-induced rabdomyolysis. Despite that, however, the quantity of various trace elements such as Zn is also changed in ESKD patients [9], it has not been investigated the effects of the difference of trace elements on statin-induced rabdomyolysis.

There is an imbalance of various trace elements, such as low plasma concentrations of Zn, in ESKD patients. In addition, patients undergoing hemodialysis (HD patients) have abnormally high plasma Cu/Zn ratios, low activity of superoxide dismutase (SOD), and high levels of oxidative stress; Zn supplementation in HD patients ameliorates Cu/Zn ratios and may reduce oxidative stress [10]. Atorvastatin is associated with mitochondrial reactive oxygen species (ROS)-induced apoptosis in glycolytic skeletal muscle and may contribute to myopathy [11]. Moreover, it has been shown that Zn deficiency induces apoptosis in a variety of tissues in rats [12], and pre-treatment with either Cu or Zn reduces growth inhibition by lovastatin in human cervical carcinoma HeLa cells [13]. Therefore, we hypothesized that the concentrations of various trace elements, such as Zn, may be changed in ESKD patients and may augment statin-induced cytotoxicity in striated muscle.

The aim of this study is to determine whether variation in the concentration of trace elements affects statin-induced cytotoxicity. Therefore, we investigated the effects of Mg, Ca, Mn, Cu, and Zn on statin-induced cytotoxicity in long-time uremic serum (US)-exposed human rhabdomyosarcoma (RD) cells.

## 2. Results

### 2.1. Simvastatin, Losartan, or Cisplatin-Induced Cytotoxicity in Serum-Exposed Cells

The survival curve for US cells was shifted to the left in response to simvastatin, and the LC_50_ value of simvastatin treated US cells was significantly decreased compared to that of normal serum (NS) cells (Table 1). In contrast, the survival curve for US cells was shifted to the right in response to losartan and cisplatin, and the LC_50_ values of US cells treated with losartan and cisplatin were significantly increased compared to those of NS cells (Table 1).

### 2.2. Simvastatin-Induced Apoptosis in Serum-Exposed Cells

**Figure 1:**
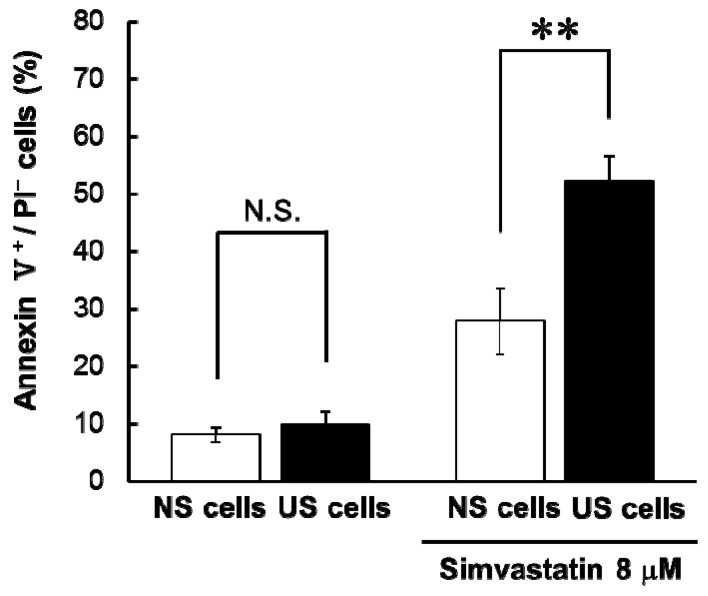
Simvastatin-induced apoptosis in serum-exposed cells. Serum-exposed cells were seeded at 1.5 × 10^5^ cells/well into six-well multiplates. After three days, the medium was changed to the differentiation medium. After seven days, the cells were incubated with the differentiation medium containing 8 µM simvastatin for 24 h. Simvastatin-induced apoptosis was determined using flow cytometry with Annexin V/propidium iodide (PI) double staining. Each column represents the mean ± S.D. (*n* = 3). Significant differences between normal serum (NS) and uremic serum (US)-exposed cells (NS and US cells) were determined using an unpaired Student’s *t*-test (** *p* < 0.01, N.S.: not significant). White column: NS cells; black column: US cells.

The number of apoptotic cells in US and NS cells was not different in the non-treatment condition, but apoptosis in US cells treated with simvastatin was greater than that in NS cells (Figure 1).

### 2.3. Intracellular Trace Elements in Serum-Exposed Cells

**Figure 2:**
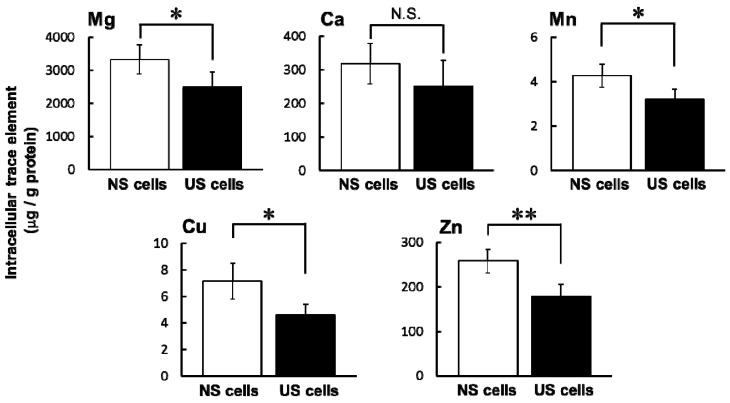
Comparison of intracellular trace elements in serum-exposed cells. Serum-exposed cells were seeded at 3.3 × 10^5^ cells/dish into a 60-mm dish. After four days, the medium was changed to the differentiation medium for seven days. Intracellular concentrations of Mg, Ca, Mn, Cu, and Zn were evaluated by inductively coupled plasma-mass spectrometry (ICP-MS). Each column represents the mean ± S.D. (*n* = 3–4). Significant differences between NS and US cells were determined using an unpaired Student’s *t*-test (* *p* < 0.05, ** *p* < 0.01, N.S.: not significant). White column: NS cells; black column: US cells.

The intracellular concentrations of Ca in NS and US cells were not different, whereas the intracellular concentrations of Mg, Mn, Cu, and Zn were significantly lower in US cells than in NS cells (Figure 2).

### 2.4. Pre-Treatment with TPEN Decreases the Level of Intracellular Trace Elements in Serum-Exposed Cells

**Figure 3:**
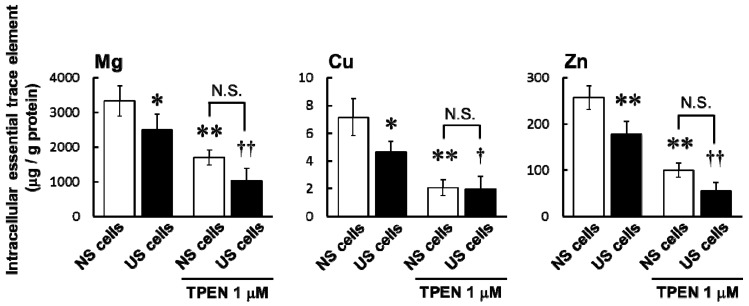
Effects of *N*,*N*,*N*′,*N*′-tetrakis (2-pyridylmethyl) ethylenediamine (TPEN) on intracellular trace elements in serum-exposed cells. Serum-exposed cells were seeded at 3.3 × 10^5^ cells/dish into 60 mm dishes. After four days, the medium was changed to the differentiation medium in the presence or absence of TPEN 1 µM and incubated for seven days. Intracellular concentrations of Mg, Cu, and Zn were evaluated by ICP-MS. Each column represents the mean ± S.D. (*n* = 3–4). Significant differences were determined by Tukey–Kramer test (* *p* < 0.05, ** *p* < 0.01 vs. untreated NS cells, ^†^ *p* < 0.05, ^††^ *p* < 0.01 vs. untreated US cells, N.S.: not significant). White column: NS cells; black column: US cells.

Intracellular concentrations of Mg, Cu, and Zn in US and NS cells were significantly decreased by pre-treatment with TPEN (*N*,*N*,*N*′,*N*′-tetrakis (2-pyridylmethyl) ethylenediamine) compared to untreated controls (Figure 3).

### 2.5. Pre-Treatment with TPEN Increased Simvastatin-Induced Cytotoxicity in Serum-Exposed Cells

**Figure 4:**
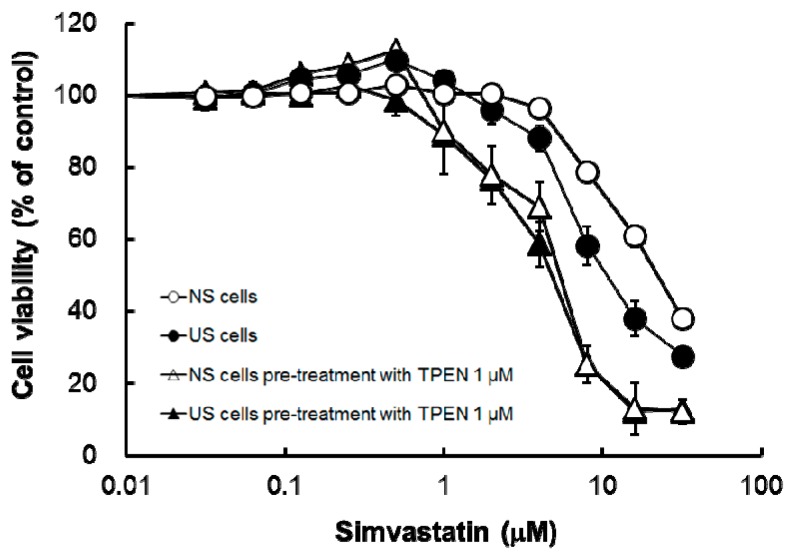
Effects of TPEN on simvastatin-induced cytotoxicity in serum-exposed cells. Serum-exposed cells were seeded at 5 × 10^3^ cells/well into 96-well multiplates. After three days, the medium was changed to the differentiation medium in the presence or absence of TPEN 1 µM. After seven days, the cells were incubated with the differentiation medium containing simvastatin at various concentrations for three days. The cytotoxicity of simvastatin was determined by CellQuanti-Blue™ Cell Viability Assay Kits. Each point represents the mean ± S.D. (*n* = 4). Open circles: NS cells; closed circles: US cells; open triangles: NS cells pre-treatment with TPEN 1 mM; closed triangles: US cells pre-treatment with TPEN 1 µM.

Pre-treatment with TPEN significantly increased simvastatin-induced cytotoxicity in both US and NS cells, and pre-treatment with TPEN suppressed the augmentation of simvastatin-induced cytotoxicity caused by US (Figure 4 and Table 2).

### 2.6. mRNA Expression of Antioxidant Enzymes and a Metal Transporter in Serum-Exposed Cells

**Figure 5:**
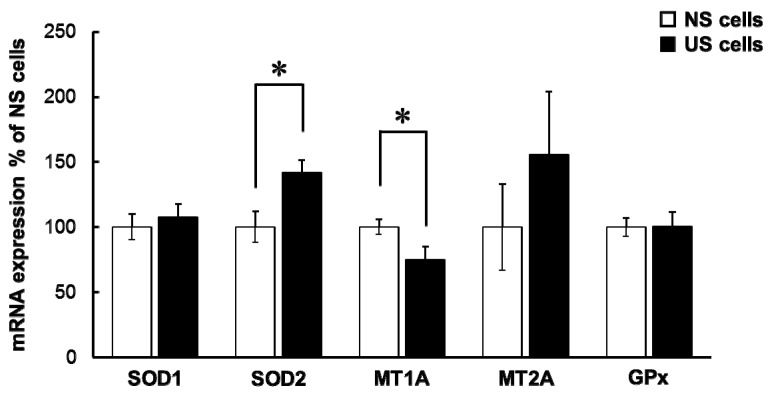
Expression of antioxidant enzymes and a metal transporter in US cells. Serum-exposed cells were seeded at 1.5 × 10^5^ cells/well into six-well multiplates. After three days, the medium was changed to the differentiation medium and incubated for seven days. The level of mRNA expression of superoxide dismutase (SOD) 1, SOD2, metallothionein (MT) 1A, MT2A, and glutathione peroxidase (GPx) was evaluated by quantitative real-time reverse transcriptase polymerase chain reaction (RT-PCR). Each column represents the mean ± S.D. (*n* = 3). Significant differences between NS and US cells were determined by unpaired Student’s *t*-test (* *p* < 0.05). White column: NS cells; black column: US cells.

The mRNA expression level of SOD2 was significantly higher in US cells than in NS cells, and that of metallothionein (MT) 1A was significantly lower in US cells than in NS cells. In contrast, the mRNA level of SOD1, MT2A, and glutathione peroxidase 1 (GPx1) were not significantly different in US and NS cells (Figure 5).

## 3. Discussion

We had previously shown that statin-induced cytotoxicity in differentiated RD cells was augmented by pre-treatment with uremic toxins—hippuric acid, 3-carboxy-4-methyl-5-propyl-2-furanpropionate, indole-3-acetic acid, and 3-indoxyl sulfate, whereas cisplatin-induced cytotoxicity was not affected by pre-treatment with uremic toxins [6]. In this study, simvastatin-induced apoptosis and cytotoxicity was greater in US cells than in NS cells (Table 1 and Figure 1), and here, we found that simvastatin-induced cytotoxicity was similarly augmented in US cells. By contrast, cytotoxicity associated with losartan and cisplatin was lower in US cells than NS cells (Table 1). The effect of long-time exposed with US on cisplatin-induced cytotoxicity in RD cells was different from effect of pre-treatment with uremic toxins [6]. It is possible that the decrease in cisplatin-induced cytotoxicity in US cells was due to serum components apart from uremic toxins. Components in US may vary sensitivity to specific drugs, and US components besides uremic toxins may also affect statin-induced cytotoxicity by accelerating drug-induced apoptosis.

One factor that may influence sensitivity to specific drugs in ESKD patients is an imbalance of various trace elements. Intracellular concentrations of Mg, Mn, Cu, and Zn were significantly lower in US cells than in NS cells (Figure 2). Ari et al. reported in HD patients that the serum levels of Zn and Mn were lower but that levels of Cu and Mg were higher compared to healthy volunteers [9]. These differences may be influenced by a meal or circadian rhythms in trace elements. A decrease in intracellular Mg increases potassium secretion by Mg-mediated inhibition of renal outer medullary potassium channel (ROMK). If ROMK in the apical membrane of distal nephron is in Mg deficiency same as RD cells, it may be one of risk factors for hypokalemia. Then, the hypokalemia can cause rhabdomyolysis [14,15].

Pre-treatment with the membrane-permeable heavy metal chelator TPEN significantly decreased intracellular concentrations of Mg, Cu, and Zn in both US and NS cells (Figure 3) and increased simvastatin-induced cytotoxicity (Figure 4 and Table 2). The result of augmentation of simvastatin-induced cytotoxicity eliminated the distinction between cytotoxicity in US and NS cells (Figure 4 and Table 2). In vivo, it has been reported that Zn is present at active centers of some antioxidant enzymes, such as SOD, and is an important factor against oxidative stress [16]. Atorvastatin was previously shown to induce changes in oxidative stress and to reduce exercise capacities in rats [17]. Therefore, deficiencies of intracellular trace elements, such as Zn, may be involved in the augmentation of oxidative stress. In important fact to HD patients, it has been reported that the serum levels of Zn is lower compared to healthy volunteers [9], and oxidative product in plasma is higher compared to healthy volunteers [10]. These results suggest that deficiencies in trace elements may also be involved in the augmentation of simvastatin-induced cytotoxicity caused by US.

mRNA levels of SOD1, MT2A, and GPx1 in US cells were comparable to that in NS cells, but the level of SOD2 mRNA in US cells was significantly higher than that in NS cells (Figure 5). In addition, mRNA levels of SOD1 were significantly decreased in NS and US cells treated with TPEN, but no changes in mRNA levels of SOD2 were found (data not shown). Therefore, augmentation of simvastatin-induced cytotoxicity in US cells may be at least partly associated with oxidative stress.

mRNA expression of MT1A in US cells was significantly lower than in NS cells (Figure 5). Metallothioneins are proteins with the capacity to bind metal, and have a function in antioxidation. Hence, alterations in simvastatin-induced cytotoxicity caused by US may be due to the low level of MT1A expression. The level of MT expression was increased by Zn and decreased by TPEN [18], suggesting that decreased levels of MT1A mRNA may be associated with intracellular Zn deficiencies.

The limitations of this study include lack of changes in oxidative stress, and the relationship between protein and mRNA expression levels. Accordingly, typical markers of oxidative stress or ROS production and the protein expression of antioxidant enzymes or a metal transporter need to be measured directly to confirm our findings.

## 4. Conclusions

In this study, we showed that the augmentation of simvastatin-induced cytotoxicity in US cells was caused, in part, by promotion of apoptosis, which resulted from trace element deficiencies such as Zn. The simvastatin-induced cytotoxicity may be alleviated partially by supplementation of trace elements such as Zn. However, further research is needed to confirm this mechanism and effect of variation in other trace elements. Our findings contribute to a growing body of evidence explaining why statin-induced rhabdomyolysis is enhanced in ESKD patients and may lead to more appropriate use of statins in these patients in the future.

## 5. Materials and Methods

### 5.1. Chemicals

Simvastatin was purchased from Toronto Research Chemicals, Inc. (North York, ON, Canada). Losartan was purchased from LKT Laboratories, Inc. (St. Paul, MN, USA). Cisplatin was purchased from Wako Pure Chemical Industries (Osaka, Japan). TPEN was purchased from Santa Cruz Biotechnology, Inc. (Dallas, TX, USA). Annexin V-fluorescein isothiocyante (FITC) was purchased from BioLegend, Inc. (San Diego, CA, USA). The Cell Quanti-Blue™ Cell Viability Assay Kit (CellQuanti-Blue™) was purchased from BioAssay Systems (Hayward, CA, USA). Pooled human serum as NS was purchased from Merck Millipore Co. (Billerica, MA, USA). Serum that was pooled from more than 400 dialysis patients as US was obtained from Shirasagi Hospital (Osaka, Japan). Because the patients were administered various drugs for medication, those drugs might affect our research. All uremic sera, however, were collected from patients just before hemodialysis. Because HD patients do not administer drugs before hemodialysis in general except for prophylactic drug for adverse events such as dialysis hypotension, it was thought that there were negligible effects of prescription drugs in US. This study (approval number 08-04) was approved in advance by the Shirasagi Hospital and Kyoto Pharmaceutical University ethical review board on 11 December 2008 and 1 April 2008, respectively.

### 5.2. Cell Culture

The RD cell line was purchased from American Type Culture Collection (Manassas, VA, USA). Dulbecco’s modified Eagle’s medium (DMEM, Life Technologies, Tokyo, Japan) without fetal bovine serum (FBS, Thermo Fisher Scientific, Inc., Kanagawa, Japan) contained sodium hydrogen carbonate (3.7 g), 100 U/mL penicillin, and 100 µg/mL streptomycin and 0.1 mM non-essential amino acids solution (Nacalai Tesque, Inc., Kyoto, Japan). NS and US were deproteinized using three volumes methanol and the supernatants were dried under a stream of nitrogen at 50 °C. The residue was dissolved in 10 volumes DMEM without FBS and ultrafiltered using a 0.22 µm membrane filter. The final concentration of 10% FBS was ultrafiltered in DMEM with serum using a 0.45 µm membrane filter, and these media were named 10% NS medium or 10% US medium, respectively. RD cells were exposed for at least one month to 10% NS medium or 10% US medium at 37 °C with 5% CO_2_. During this period, RD cells were re-plated at a density of 1 × 10^6^ cells per 10 mL in media every third or fourth day. The resulting cells were named NS and US cells, respectively.

### 5.3. Evaluation of Cytotoxicity

RD cells that grown as the following are a model cells of human skeletal muscle and used for myotoxicity evaluation in this study [19]. NS and US cells were plated at a density of 5 × 10^3^ cells/well/100 µL into 96-well plates in DMEM with 10% FBS for three days at 37 °C with 5% CO_2_. These cells were then cultured in DMEM with 1% FBS (differentiation medium) for seven days to induce differentiation. In the pre-treatment experiment with TPEN, 1 µM TPEN was added to the differentiation medium. After removal of the medium from differentiated RD cells, differentiated RD cells were exposed to medium containing the following test compounds for three days at 37 °C with 5% CO_2_. The concentrations of simvastatin, losartan, and cisplatin (test compounds) used were 0.03125–32 or 0.25–256 µM, 16–4096 µM, and 0.5–512 µM, respectively.

After treatment with the test compounds for three days, cell viability was measured using CellQuanti-Blue™ and a microplate reader (excitation wavelength = 535 nm, emission wavelength = 590 nm, GENios, Tecan, Seestrasse, Switzerland).

The lethal concentration 50 (LC_50_) value was calculated using the non-linear least squares program (MULTI)

$$L\;=\;L_{\max}\times \;(1-\frac{C^{\gamma }}{C^{\gamma }+L{C_{50}}^{\gamma }})$$

where *L*, *L*_max_, *C*, and γ are cell viability (% of control), maximum cell lethality, drug concentration in the medium, and sigmoid function, respectively.

### 5.4. Assessment of Apoptosis

NS and US cells were incubated in medium containing 8 µM simvastatin for 24 h (shorter than cytotoxicity experiment) at 37 °C with 5% CO_2_. NS and US cells dissociated by trypsin-EDTA were suspended at a concentration of 1 × 10^6^ cells/mL in ice-cold buffer (140 mM NaCl, 2.5 mM CaCl_2_, 10 mM 2-[4-(2-hydroxyetyl)-1-piperazinyl] ethanesulfonic acid (HEPES)/NaOH, pH 7.4). Annexin V-FITC (5 μL) and 50 µg/mL propidium iodide (PI) solution (1 μL) were added to the 100 μL cell suspension and incubated for 15 min in the dark. The reaction solution was diluted five-fold in the same buffer and then analyzed using fluorescence activated cell sorting (FACS) Calibur™ (Becton Dickinson, Franklin Lakes, NJ, USA). Apoptotic cells were defined as those that were annexin V-FITC-positive and PI-negative.

### 5.5. Measurement of Intracellular Trace Elements

NS and US cells were lysed by addition of 1% sodium dodecyl sulfate (5 mL). After pouring the cell lysate (4.7 mL) into a nitric acid-treated tall beaker, the lysate was evaporated completely on a hot plate at 170 °C. Upon addition, in turn, of nitric acid, perchloric acid, and hydrogen peroxide to the residue, the residue was dried under the same conditions as described above. Three cycles of the same operation were performed for complete incineration of the sample.

The incinerated sample was lysed by addition of nitric acid (9 mL), and the concentrations of intracellular Mg, Ca, Mn, and Zn were determined by inductively coupled plasma-mass spectrometry (ICP-MS, Agilent 7700, Agilent Technologies Japan, Ltd., Tokyo, Japan). Prepared calibration standard samples were 0, 5, 10, 50, 100, and 500 ppb. The overall relationship among the standards was best described by a linear relationship (*r*^2^ > 0.999).

### 5.6. Measurement of mRNA

NS and US cells were lysed by addition of RNAzol^®^RT Reagent (1 mL, Molecular Research Center, Inc., Cincinnati, OH, USA). After adding diethyl pyrocarbonate-treated water (400 µL) to the cell lysates, they were vortex mixed for 15 s, left at room temperature for 15 min, and centrifuged at 12,000× *g* at 25 °C for 15 min. After adding isopropanol (1 mL) to the supernatant (1 mL), the supernatant was left at room temperature for 10 min. RNA precipitate formed a pellet upon centrifugation at 12,000× *g* at 25 °C for 15 min. The residue was removed from the supernatant and was washed twice using 75% ethanol (500 µL). After washing, RNase-free water (50 µL) was added to the residue, and the RNA in the residue was quantified spectrophotometrically at 260 nm using a DU^®^730 (Becton Dickinson). RNA quantity was calculated using the formula

RNA quantity (µg/mL) = A_260_ × 40

The RNA solutions were diluted to 100 µg/mL with RNase-free water and stored at −80 °C.

Reverse transcriptase polymerase chain reaction (RT-PCR) was performed using ReverTra Ace^®^ qPCR (Toyobo, Ltd., Osaka, Japan). For preparation of cDNA, the total reaction volume per sample was 20 µL (9 µL of nuclease-free water, 4 µL of 5× RT buffer, 1 µL of RT enzyme mix, 1 µL of primer mix, 5 µL of RNA). RT-PCR was performed using an i-Cycler iQ (Bio-Rad Laboratories, Inc., Hercules, CA, USA) with 37 °C for 15 min (reverse transcription reaction), 98 °C for 5 min (inactivation of reverse transcriptase), and then 4 °C (cooling). DNA was amplified in a total reaction solution volume of 20 µL (7.16 µL of sterile distilled water, 10 µL of Thunderbird^TH^SYBR^®^ qPCR Mix (Toyobo), 0.4 µL of 10 µM sense primer, 0.4 µL of 10 µM antisense primer, 0.04 µL of 50× ROX reference dye (Toyobo), 2 µL of RT sample). Real-time PCR was performed using a LightCycler^®^ Nano System (Roche Diagnostics K.K., Tokyo, Japan). The initial denaturation was 95 °C for 1 min and was followed by 45 cycles of amplification, with a thermal cycling profile of 95 °C for 10 s and 60 °C for 30 s. Beta-2-microglobulin (β2M) was used as a housekeeping gene. Primer sequences are specified in Table 3.

C_T_ was calculated using the fluorescence intensity of SYBR^®^ Green I, and the amount of target mRNA relative to β2M mRNA was expressed as 2^−^^⊿CT^, where ⊿C_T_ is the value obtained by subtracting the C_T_ value of β2M mRNA from the C_T_ value of the target mRNA.

### 5.7. Statistical Analysis

Measured values and LC_50_ values were expressed as mean ± standard deviation (S.D.) and median (95% confidence intervals), respectively. Non-overlapping confidence intervals of LC_50_ were considered statistically significant. The significant differences between groups were determined using unpaired Student’s *t*-test or non-repeated measures analysis of variance (ANOVA) followed by Tukey–Kramer multiple comparison test. A *p* value less than 0.05 was considered statistically significant.

## Figures and Tables

**Table 1:** Lethal concentration 50 (LC_50_) values for simvastatin, losartan, and cisplatin in serum-exposed cells.

Cell Type	LC_50_ Value (95% Confidence Interval)		
	Simvastatin (µM)	Losartan (µM)	Cisplatin (µM)
NS cells	6.15 (5.78–6.73)	582 (569–596)	51.1 (48.2–54.0)
US cells	3.67 (3.19–4.15) *	764 (735–793) *	64.2 (60.6–67.9) *

Non-overlapping 95% confidence intervals; * vs. NS cells; Data determined by CellQuanti-Blue™ Cell Viability Assay Kits represent the mean (*n* = 4).

**Table 2:** LC_50_ values for simvastatin in serum-exposed cells with or without TPEN

Cell Type	LC_50_ Value (95% Confidence Interval)
	Simvastatin (µM)
NS cells	21.61 (20.50–22.71)
NS cells + TPEN 1 µM	4.75 (4.06–5.43) *
US cells	11.25 (10.11–12.39) *
US cells + TPEN 1 µM	4.46 (4.09–4.84) ^†^

Non-overlapping 95% confidence intervals; * vs. NS cells; ^†^ vs. US cells; Data determined by CellQuanti-Blue™ Cell Viability Assay Kits represent the mean (*n* = 4).

**Table 3:** Sequences of primers used to amplify different genes by real-time PCR.

Gene (PCR Products Size)	Sequences
SOD1 (174 b.p.)	Sense 5′-GAA GGT GTG GGG AAG CAT TA-3′ Antisense 5′-ACA TTG CCC AAG TCT CCA AC-3′
SOD2 (313 b.p.)	Sense 5′-CGT CAC CGA GGA GAA GTA CC-3′ Antisense 5′-CTG ATT TGG ACA AGC AGC AA-3′
MT1A (156 b.p.)	Sense 5′-ACT GGT GGC TCC TGC ACC TGC ACT-3′ Antisense 5′-ACA GCA GCT GCA CTT CTC TGA T-3′
MT2A (259 b.p.)	Sense 5′-CCG ACT CTA GCC GCCTCT T-3′ Antisense 5′-GTG GAA GTC GCG TTC TTT ACA-3′
GPx1 (85 b.p.)	Sense 5′-CCA AGC TCA TCA CCT GGT CT-3′ Antisense 5′-TCG ATG TCA ATG GTC TGG AA-3′
β2M (313 b.p.)	Sense 5′-TGC TCG CGC TAC TCT CTC TTT-3′ Antisense 5′-TTC TCT GCT TGA CGT GAG TAA-3′
